# Lessons Learned from Replicating a Randomized Control Trial Evaluation of an App-Based Sexual Health Program

**DOI:** 10.3390/ijerph18063305

**Published:** 2021-03-23

**Authors:** Jennifer Manlove, Brooke Whitfield, Jane Finocharo, Elizabeth Cook

**Affiliations:** Child Trends, 7315 Wisconsin Avenue, Suite 1200W, Bethesda, MD 20814, USA; bwhitfield@childtrends.org (B.W.); jfinocharo@childtrends.org (J.F.); ecook@childtrends.org (E.C.)

**Keywords:** sexual health program, app, randomized controlled trial, Black, Latinx, sexual and reproductive health, replication study

## Abstract

This study presents findings from a randomized control trial replication evaluation of Pulse, an app-based pregnancy prevention program implemented with Black and Latinx women aged 18–20, a population with high rates of unplanned pregnancy. We used social media advertisements to enroll 1013 women online across the U.S. and automatically randomized participants to either the Pulse reproductive health app or a general health control app, stratifying by age and race/Latinx ethnicity. Participants received reminder text messages to view the app as well as text messages with app-related content throughout the intervention. Linear probability models were conducted on the analytic sample of 871 participants who completed the six-week survey and 798 who completed the six-month survey and adjusted for permuted block randomization and multiple hypothesis testing. Compared to the control group, intervention group participants had higher contraceptive knowledge (*p* = 0.000), which replicates findings from an earlier evaluation. However, these impacts were not sustained at six-month follow-up (*p* = 0.162). We found no other significant program impacts. This contrasts with an earlier evaluation that found intervention participants were less likely to have had sex without a hormonal or long-acting reversible contraceptive (LARC) method and had greater self-confidence to use contraception consistently than the control group. Different demographic characteristics, lower app usage, and more negative attitudes about and usage of hormonal/LARC contraception in the current sample may help to explain fewer impacts than the earlier evaluation.

## 1. Introduction

Teen pregnancy prevention (TPP) programs are increasingly incorporating technological components or are implementing entirely technology-based interventions. Technology-based programs offer several advantages. For instance, they can be more cost effective than traditional interventions [1,2], and they can be implemented with high fidelity, because all content is pre-programmed with accurate information and delivered identically to all participants [1,3]. They also have the potential to reach populations who are not typically served by classroom-based programs, such as older teens (ages 18–19), who account for 75 percent of births to mothers aged 15–19 and experience high rates of unintended pregnancy [1,4,5]. Additionally, other research has found that technology-based interventions and access to websites or apps with sexual and reproductive health content may have positive impacts on outcomes for young women, such as increased knowledge of sexually transmitted infections (STIs) and reduced rates of unprotected sex and unintended pregnancy [1,6,7,8,9,10]. However, very few evidence-based teen pregnancy prevention programs are technology-based [11], highlighting the need to expand research and evaluation in this area.

There is also a need for more research on programs tailored to meet the needs of Black and Latinx teens, who have historically been underserved by sexual and reproductive health efforts. In addition to being underserved, many Black and Latinx teens experience racial bias when they do receive contraception counseling [12,13] and consequently report lower levels of trust in healthcare providers and family planning efforts [14,15]. This likely contributes to the fact that Black and Latinx teens aged 15–19 experience birth rates that are approximately 50 percent higher than the national average for 15–19-year-olds in the United States [4].

Some previous research has found that technology-based programming is relevant for Black and Latinx teens [16,17,18]. For example, a previous evaluation of Pulse, an app-based sexual and reproductive health intervention implemented with women aged 18–20 who were recruited online, found several promising preliminary impacts. This evaluation enrolled 1304 women between November 2016 and March 2018, 76 percent of whom were Black or Latinx. At six-week follow-up, Pulse participants were significantly less likely to report having had sex without a hormonal or long-acting reversible contraceptive (LARC) method, had higher contraceptive knowledge, and were more confident they could use contraception every time they have vaginal intercourse compared to the control group [16].

This paper presents the results of a replication of the evaluation of Pulse with a more recent sample. Replications of evaluations that have found positive impacts can help to expand the knowledge base related to teen pregnancy prevention, as few evaluations of evidence-based TPP programs have been replicated [19,20]. Replications can evaluate the efficacy of programs when implemented in a new setting, with a different population, or on a larger scale [19,21], and can help to assess programs’ relevance and effectiveness as time passes and the context in which they are implemented shifts [19]. The objective of this replication study is to extend previous research by (1) testing short-term impacts of Pulse among Black and Latinx women aged 18–20, who have limited access to sexual and reproductive health services and high rates of unintended teen pregnancy [4,22]; (2) assessing longer-term impacts on behavioral outcomes (pregnancy, receipt of sexual and reproductive health services) with a six-month follow-up survey not included in the original study; and (3) comparing similarities and differences between this replication evaluation and the earlier evaluation on impacts, as well as differences in sample characteristics. Given the original study’s short-term impacts on unprotected sex, knowledge, and self-efficacy, we hypothesized that the Pulse intervention would have similar short-term impacts for the replication study. We also hypothesized that these impacts would persist at six-month follow-up and that we would find additional impacts on behavioral outcomes.

## 2. Materials and Methods 

We conducted a randomized control trial (RCT) evaluation, incorporating continuous online recruitment, individual-level random assignment, and online survey data collection with text-based notifications. The study was conducted between October 2018 and November 2019. The Child Trends Institutional Review Board approved the study (IRB protocol number 1369.00.003).

### 2.1. Study Procedures

#### 2.1.1. Enrollment and Randomization

Individuals met the study eligibility criteria if they were female, aged 18–20, lived in the United States or a U.S. territory, were Latinx or Black, were not pregnant or trying to become pregnant, had daily access to a smartphone, and spoke English (because the app was developed in English) at the time of enrollment. Anyone who did not meet all eligibility criteria was excluded. We offered Amazon electronic gift cards as incentives for enrolling in the study and completing surveys. 

**Figure 1:**
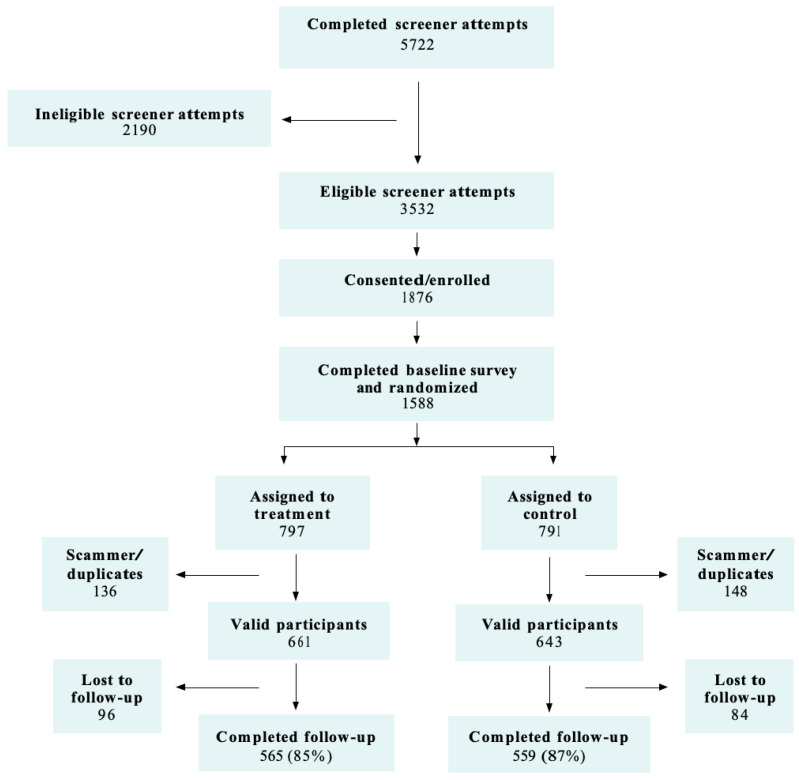
Pulse participant flow diagram.

The study enrolled 1013 participants into the replication study (see Figure 1) using social media advertisements. After clicking on a recruitment ad, individuals were sent to a web page with an eight-question screener to assess whether the individual was eligible for the study. There were 5553 completed screener attempts, 47 percent of which were from eligible respondents (*n* = 2619). Of the eligible individuals, 1397 completed an enrollment and consent form, and 1204 completed the 40-question baseline survey. After completing this process, participants were immediately randomized and sent to the registration page of either the Pulse intervention app or the general health control app. To ensure equivalence across the intervention and control groups, the study used a permuted block design with stratifiers for age at enrollment (18, 19, or 20) and race/Latinx ethnicity (Latinx or non-Latinx Black).

#### 2.1.2. Scammers and Duplicate Accounts

Since recruitment occurred entirely online, the study was susceptible to enrolling ineligible participants such as scammers (ineligible individuals who completed the screener multiple times until they were eventually eligible) and duplicates (eligible individuals who enrolled in the study more than once). To ensure we only enrolled people who met the recruitment criteria, we developed detailed procedures to identify and remove scammers and duplicate accounts from the sample. We removed 191 ineligible accounts from the 1204 that were randomized, for a final sample of 1013 participants.

#### 2.1.3. Intervention and Control Apps

The Pulse app was designed by Healthy Teen Network to provide sexual and reproductive health content for Latinx and Black young women aged 18–20 [16]. The Pulse design team incorporated input from Black and Latinx teens to inform app content and multimedia related to accessing health services, use of birth control, and birth control attitudes and beliefs. Pulse is grounded in the Theory of Planned Behavior and Self-Efficacy, as well as Social Learning Theory [23,24]. The app is self-led and does not require users to follow a specific sequence of content. Moreover, users can access the app anywhere on their mobile device with internet connection and can interact with Pulse as frequently or infrequently as they choose during the six-week intervention period. 

Pulse provides comprehensive, medically accurate sexual and reproductive health information to young women to help users to choose an effective birth control that meets their needs, access reproductive health services, and prevent unintended pregnancies. Pulse has six interactive sections covering approximately three hours of material related to birth control methods, healthy relationships, sexual consent, anatomy and physiology pregnancy, sexually transmitted infections, and clinic access (including a clinic locator). Each section includes engaging activities, such as appointment reminders and videos modeling real-life scenarios like clinic visits [25,26].

Participants in the control condition received access to a general health app also created by Healthy Teen Network. The control app has a design that is similar to that of Pulse, but the control app contains no information about reproductive health and instead focuses on topics such as exercise, healthy eating, sleep, and emotional health. 

#### 2.1.4. Text Messages

Approximately every three days, intervention and control group participants received pre-programmed text messages which included app-related content and highlighted app activities. Additionally, all participants received reminder text messages to view the app and complete the follow-up surveys.

### 2.2. Data Collection

Participants took the baseline survey before randomization. Once randomized, participants who registered with the app received their first incentive (a $25 Amazon electronic gift card) via email from the study team. Six weeks post-randomization, we sent participants a link to the short-term follow-up survey. To encourage participants to complete the survey, we sent reminder text messages and called participants to follow up if they had not completed the survey. Participants had one month to take the survey and received their second incentive (a $20 Amazon gift card) upon completion. Eighty-six percent of intervention participants (434 of 504) and 86 percent of the control participants (437 of 509) completed the short-term follow-up survey, indicating minimal differential attrition. We followed the same procedure for the six-month follow-up survey, and participants received their third incentive (a $25 gift card) upon completion. Eighty percent of intervention participants (402 of 504) and 78 percent of control participants (396 of 509) completed the six-month follow-up survey, which also indicates minimal differential attrition.

### 2.3. Survey Instruments and Measures

As in the original study, we incorporated survey items from several sources, including national surveys [27,28,29], other federally funded teen pregnancy prevention evaluations [10,30,31], required measures from the Office of Population Affairs [32], and measures designed and tested by the evaluation team [16]. 

The primary and secondary short-term behavioral outcomes for this replication study were the same as the original study. The primary outcomes include (1) sexual intercourse without using any method of contraception and (2) sexual intercourse without a hormonal contraceptive (birth control pills, the shot, the patch, the ring) or LARC (intrauterine device or implant). Each of these items were assessed during the last six weeks (for the short-term follow-up) and the last three months (for the six-month follow-up). The secondary outcome measures include (1) currently using a hormonal contraceptive or LARC method and (2) hormonal or LARC use during last sex for a subpopulation of participants who were sexually active at baseline. 

In addition to these short-term outcomes of interest, this replication study included three additional long-term secondary outcomes of interest: (1) ever been pregnant, (2) experienced a pregnancy scare in the past six months, and (3) visited a provider for sexual and reproductive health services in the past six months. These long-term outcomes are unique to this replication study since the original study did not include a six-month follow-up and therefore was unable to measure these secondary outcomes of interest.

The study also included secondary outcomes measuring knowledge, attitudes, self-efficacy, and intentions related to sexual and reproductive health. These include a four-item measure of birth control knowledge (reflecting the percentage of items related to birth control effectiveness that were answered correctly), and two items measuring attitudes about birth control, based on whether participants disagreed that “birth control is too much of a hassle to use” and “birth control has too many negative side effects” (compared to those who either agreed or neither agreed nor disagreed). Two items assess attitudes toward sexual and reproductive health services, based on whether participants disagreed that “going to a health care provider for sexual and reproductive health services is hard” and “going to a health care provider for sexual and reproductive health services is expensive” (compared to those who agreed or neither agreed nor disagreed).

An indicator of birth control self-efficacy measures whether participants agreed (vs. disagreed or neither agreed nor disagreed) with the statement, “I am confident that I can use birth control every time I have sex.” Sexual and reproductive health self-efficacy measures whether participants agreed (vs. disagreed or neither agreed nor disagreed) with the statement, “I am confident that I can go to a health care provider for sexual and reproductive health services.” Finally, two items assess intentions, based on whether participants responded that they definitely “plan to visit a health care provider (clinic or doctor’s office) for sexual or reproductive health services in the next 12 months” and “intend to use one of the following methods” (followed by a list of hormonal/LARC methods) if they were to have vaginal intercourse in the next year (compared to those who responded: Yes, probably; No, probably not; No, definitely not; or Don’t know). 

### 2.4. App Usage

We analyzed participant app usage to assess dosage. These data were downloaded from the app’s website using the SlimStat plugin on the WordPress dashboard. Text messaging data from the EZ Texting platform were used to assess participant receipt of text messages and whether participants opted out of receiving app-related texts.

### 2.5. Analysis 

We conducted *t*-tests with adjustments for permuted block random assignment to determine baseline equivalence between intervention and control groups and assess differences in participants’ sociodemographic characteristics, sexual and reproductive attitudes and behaviors, and app usage between the original and replication studies. The multivariate impact analyses incorporated an intention-to-treat approach, and used adjusted *p*-values to account for multiple hypothesis testing [33]. We also incorporated clustered standard errors in the impact analyses to adjust for the permuted block random assignment [34].

We used linear probability models [35] to assess the impact of Pulse on each outcome of interest, conducting short-term impact analyses with the sample of 871 participants who completed the six-week follow-up survey and long-term analyses with the analytic sample of 798 participants who completed the six-month follow-up. All analyses controlled for sociodemographic characteristics (age at baseline and race/Latinx ethnicity), sexual experience (ever had vaginal sex at baseline), and the outcome of interest, measured at baseline. We also conducted supplemental sensitivity analyses (available on request from the lead author), incorporating additional covariates and removing the random block design control from the models. All analyses were completed using Stata 16.1 [36].

## 3. Results

Table 1 compares the baseline characteristics for the intervention group and control group samples who completed the six-week follow-up survey. No statistically significant differences were found in attrition rates by treatment group. The intervention and control groups did not significantly differ on any sociodemographic or behavioral outcome measure. Baseline characteristics for the six-month analytic sample (not shown) indicate that intervention participants were significantly less likely to have had sex without a hormonal or LARC method than control participants (28 vs. 34 percent). 

Table 2 presents the sociodemographic characteristics and app usage data of intervention participants in the original and replication studies who completed the six-week follow-up survey. This allows us to examine differences between the two study populations. The average age of both samples was approximately 19 years, and most participants (76–80 percent) reported living with family. Both samples reported similar percentages of having at least one child (6–9 percent) and had similar sexual histories at baseline. Two thirds of the original and replication study samples had ever had vaginal sex (67–69 percent), and over half had had sex in the past three months (55–57 percent). Approximately 1 in 10 (9–12 percent) reported having ever been pregnant and nearly half (49 percent) reported ever having a pregnancy scare. At baseline, approximately one quarter of participants (24–26 percent) reported having sex without using any method of contraception and 28–29 percent had had sex without using a hormonal or LARC method—measured in the past three months.

Table 2 also shows significant differences between the original and replication study samples across multiple measures. The replication study included only Latinx and non-Latinx Black participants, while almost one quarter of the original sample were neither Black nor Hispanic. These non-Hispanic Other participants identified as White, Asian, American Indian, Native Hawaiian/Pacific Islander, and Other. Although replication study participants had high educational attainment (62 percent completed at least some college or technical school education), they had lower levels of education than the original study sample. Replication study participants were also less likely to currently be using a hormonal or LARC method (40 vs. 49 percent) and to have used a hormonal or LARC method at last sex (47 vs. 58 percent). Replication study participants had more negative attitudes toward birth control use than the original sample and lower levels of birth control self-efficacy. Replication study participants also had significantly lower levels of app usage than the original study sample. Replication study participants were less likely to log into the app more than once, had fewer average numbers of logins, fewer total clicks within the app, and visited fewer sections than participants in the original study sample. However, replication study participants were more likely to receive study related text messages.

Table 3 presents six-week impact results for the original and replication study participants who completed the follow-up survey. Similar to the original study, the replication study found a significant difference between the intervention and control groups on the secondary outcome measure of birth control knowledge. Intervention participants in the replication study had greater birth control knowledge (50 vs. 42 percent; *p* = 0.000) than control participants in multivariate analyses. However, in contrast to the original study, the replication study did not find significant impacts on the primary outcome measure of sex without a hormonal or LARC method.

Similar to the original study, the replication study did not demonstrate impacts on the second primary outcome measure of sex without any contraceptive method. The replication study also did not find impacts on any secondary outcomes (current hormonal/LARC use, attitudes, self-efficacy, or intentions) aside from knowledge. The original study found positive impacts on self-efficacy of using birth control during every sexual intercourse (*p* = 0.025) [16], which was not replicated in the current study.

As indicated in Table 4, the replication study’s impacts on knowledge were not sustained at the six-month follow-up (*p* = 0.162). The replication study did not find significant differences between the intervention and control groups on either of the primary outcome measures of unprotected sex or any other secondary outcome measures, including having ever been pregnant, experiencing a pregnancy scare in the past six months, or visiting a provider for sexual and reproductive health services in the past six months.

## 4. Discussion

We conducted an online RCT replication evaluation of the Pulse sexual health app with Black and Latinx women aged 18–20 in 2018–2019. However, this study did not replicate the behavioral impacts of the original evaluation (conducted in 2016–2018) on sex without a hormonal or LARC method [16]. Neither study found differences between the intervention and control groups in rates of recent sex without the use of any contraceptive method. The only other significant impact of this evaluation was increased knowledge about contraceptive methods and effectiveness at the six-week follow-up, which was also found in the original study. This evaluation extended previous evaluation findings by including a six-month longer-term follow-up; however, there were no impacts on any of the longer-term behavioral outcomes (pregnancy, clinic visit to receive sexual and reproductive health services, or unprotected sex).

Differences in the sample characteristics of the two studies may help to explain their differing impacts. This replication study sample comprised only Black and Latinx women, while one quarter of the original study sample included women who did not identify as Black or Latinx. Prior research has found that, when deciding on a contraceptive method, Black and Latinx women are more likely than white women to prioritize protection against sexually transmitted diseases, continued menstruation, control over whether and when to use the method, and ability to become pregnant shortly after stopping use, indicating a desire to preserve control over their reproduction [37]. These contraceptive preferences may be due to historical and current reproductive abuse of women of color and help to explain why Black and Latinx women overall are less likely to rely on hormonal or LARC methods compared to white women in the United States [37]. Black and Latinx women also have higher levels of mistrust in the United States medical system [14,15], which may also stem from the historical medical exploitation of these groups and the lower standard of care they currently receive [12,13,15]. These higher levels of mistrust can discourage women from choosing hormonal or LARC methods, which require more interaction and dependency on medical providers for insertion and removal [22,38].

Thus, the race/ethnic mix of the study samples may account for differences in their attitudes about and use of birth control and ultimately the effectiveness of the app. At baseline, the current replication study sample had lower levels of education, lower levels of current or recent hormonal or LARC use, more negative attitudes about birth control, and lower birth control self-efficacy than the original study’s baseline sample. Because the Pulse app aims to virtually link women to services they need—such as locating clinics, arranging appointments, or obtaining prescriptions—the app may be more relevant and effective for women who have more positive attitudes toward hormonal or LARC methods, thus being more likely to use these contraceptive methods [29].

The lower level of app usage in the current replication sample, as compared to the original sample, may have also contributed to the differences in impacts. For example, the replication sample participants visited fewer app sections, completed fewer activities, and were less likely to log into the app more than once compared to the original sample. Other research has found that lower dosage in online interventions is linked to fewer program impacts [39]. The lower app usage among the replication sample may be due, in part, to differences in population characteristics; for example, separate analyses found that having more negative attitudes about contraception was linked to using the app less [40].

Lower app usage in the replication study may also be due to the timing of recruitment. The original study sample was recruited between November 2016 and January 2018, and the replication sample was recruited more than one year later, between October 2018 and March 2019. During this more recent time period, the news media highlighted a Facebook data breach by a research firm [41], which may have exacerbated concerns about privacy in social media studies [42]. Despite these issues, the evaluation study successfully recruited a sample of more than 1000 women through social media. However, as social media recruitment becomes more prevalent [43,44], participants recruited online may be less motivated to spend large amounts of time on a single study.

While online recruitment, data collection, and the implementation approach for this evaluation were cost effective and allowed us to reach a large number of Black and Latinx women in a narrow age group, this approach may have also negatively affected participants’ feeling of being connected to the program. This could explain the low level of app usage in both the current replication study and the original study, in comparison to in-person technology-based interventions [45]. As a result, some participants (15 percent in this study) never logged into the app, and only 4 in 10 participants viewed content in all six of the app’s sections. In contrast, other evaluations incorporated more controlled conditions for participants. For example, an evaluation of a completely computer-based pregnancy prevention program that was delivered in a school-based setting found high dosage among treatment group youth [39]. Future research and implementation could explore tactics for increasing app usage, for example, by providing the Pulse app in a school-based or clinic-based setting, and/or in combination with in-person instruction. Additionally, providing a Spanish-language version of the Pulse app could reach a broader population and an important demographic since the app was designed for Latinx women.

Further, Pulse was designed primarily for cisgender, heterosexual women. Based on input from Black and Latinx teens, Pulse was subsequently updated with LGBTQ+ inclusive language. However, Pulse does not fully address or meet the needs of LGBTQ+ women. This is particularly important for future reproductive health interventions to address, since the proportion of people who report being LGBTQ+ is rising. In 2018, the General Social Survey (GSS) found that 23 percent of Black women aged 18–34 identified as bisexual—a proportion nearly three times higher than in 2008 [46]. To meet the needs of young women and fully understand evaluation findings, future reproductive health interventions should be LGBTQ+ inclusive and understand participants’ sexual orientations and gender identities.

## 5. Conclusions

This evaluation study was successful in using social media to recruit a sample of Black and Latinx women aged 18–20 to participate in an exclusively technology-based pregnancy prevention program. Young adult Black and Latinx women (ages 18–24) experience higher pregnancy rates than young adult women overall in the United States, and the majority of pregnancies among this age group are unintended [4,5]. Most of the study participants were also out of high school and therefore less likely to be receiving pregnancy prevention programming [47]. Behavioral impacts from the original evaluation of Pulse were not present in this replication study. Compared to the original evaluation that included women who did not identify as Black and/or Latinx, participants in this replication study were only Black and/or Latinx. These participants had more negative attitudes about and lower usage of effective contraceptive methods, on average at baseline, and used the app less.

This study had several strengths, including effective online recruitment of a large sample of Black and Latinx women, incorporating an RCT design, and strong response rates at both follow-ups. These strengths were counter-balanced, in part, by low app usage among participants. Program developers should continue to co-create digital interventions with young adults to ensure that the method of delivery is relevant, and that young adults consume the digital content. These interventions should tailor sexual and reproductive health programming to address unique issues for Black and Latinx women, including sexual orientation/gender identity, contraceptive preferences that may be rooted in historical and current experiences of racism, and distrust of the healthcare system.

## Figures and Tables

**Table 1:** Baseline characteristics of replication study participants who completed the six-week follow-up survey by intervention/control status (*n* = 871).

Measure	Pulse Intervention %/Mean	Control %/Mean	Difference	*p*-Value *
**Sociodemographic characteristics**				
Age at baseline (mean years)	18.7	18.8	0.0	0.656
Race/Hispanic ethnicity				
Hispanic	53.5%	49.2%	−4.3%	0.209
Non-Hispanic Black	46.5%	50.8%	4.3%	0.209
Highest level of education attained				
Less than high school degree or GED	19.8%	24.0%	4.2%	0.133
High school degree or GED	18.0%	14.0%	−4.0%	0.106
Some college, technical school, or more	62.2%	61.8%	−0.4%	0.897
Has at least one child	8.8%	8.7%	−0.1%	0.967
Currently living with family	76.0%	79.2%	3.1%	0.267
**Sexual activity and pregnancy history**				
Ever had vaginal sex	66.9%	66.1%	−0.8%	0.793
Vaginal sex in the past three months	54.6%	54.9%	0.3%	0.926
Ever been pregnant	12.1%	12.1%	0.0%	0.983
**Unprotected sex (in past 3 months)**				
Sex without any method	26.2%	29.0%	2.8%	0.361
Sex without a hormonal/LARC method	28.5%	33.4%	4.9%	0.122
**Contraceptive use**				
Current hormonal/LARC use ^a^	40.3%	36.1%	−4.2%	0.304
Hormonal/LARC use at last sex ^b^	47.2%	41.0%	−6.2%	0.173
**Sample N**	434	437		

^a^ “Current hormonal/LARC use” is measured for the 576 participants who had ever had sex. ^b^ “Hormonal/LARC use at last sex” is measured for the 474 participants who had had sex in the past three months. * *p*-values < 0.05. LARC: long-acting reversible contraceptive.

**Table 2:** Differences in baseline characteristics and usage data of intervention group participants who completed the six-week follow-up, for original study [16] and current replication study.

Measure	Original Study %/Mean	Replication Study %/Mean	Difference	*p*-Value *
**Sociodemographic characteristics**				
Age at baseline (mean years)	18.8	18.7	0.0	0.679
Race/Hispanic ethnicity				
Hispanic	38.8%	53.5%	14.7%	0.000 *
Non-Hispanic Black	37.3%	46.5%	9.2%	0.003 *
Non-Hispanic Other ^a^	23.9%	0.0%	−23.9%	0.000 *
Highest level of education attained				
Less than high school	16.6%	19.8%	3.2%	0.195
High school degree or GED	11.7%	18.0%	6.3%	0.005 *
Some college, technical school, or more	71.7%	62.2%	−9.5%	0.002 *
Has at least one child	6.4%	8.8%	2.4%	0.153
Currently living with family	80.2%	76.0%	−4.1%	0.115
**Sexual activity and pregnancy history**				
Ever had vaginal sex	68.8%	66.9%	−2.0%	0.513
Vaginal sex in the past three months	56.6%	54.6%	−1.9%	0.544
Ever been pregnant	8.9%	12.1%	3.2%	0.100
Ever had a pregnancy scare	48.7%	49.0%	0.3%	0.928
**Unprotected sex (in past 3 months)**				
Sex without any method	23.5%	26.2%	2.7%	0.331
Sex without a hormonal/LARC method	28.3%	28.5%	0.2%	0.946
**Contraceptive use**				
Current hormonal/LARC use ^b^	49.4%	40.3%	−9.1%	0.019 *
Hormonal/LARC use at last sex ^c^	58.2%	47.2%	−10.9%	0.011 *
**Birth control knowledge (% correct)**	43.4%	39.7%	−3.7%	0.092
**Attitudes**				
**Attitudes about birth control**				
Disagree that birth control is too much of a hassle to use	59.8%	52.0%	−7.8%	0.014 *
Disagree that birth control has too many negative side effects	37.8%	29.4%	−8.5%	0.005 *
**Attitudes about sexual and reproductive health care**				
Disagree that going to a health care provider for sexual and reproductive health services is hard	47.7%	50.1%	2.4%	0.448
Disagree that going to a health care provider for sexual and reproductive health services is expensive	25.6%	25.8%	0.1%	0.961
**Self-Efficacy and Intentions**				
**Self-efficacy to use birth control**				
Confident can use birth control during every sexual intercourse	63.9%	56.1%	−7.9%	0.012 *
**Self-efficacy to access sexual and reproductive health services**				
Confident can go to a health care provider for sexual and reproductive health services	78.4%	79.9%	1.5%	0.573
**Intentions**				
Intend to visit a health care provider for sexual or reproductive health services	37.8%	36.7%	−1.0%	0.735
Intend to use a hormonal/LARC method	58.3%	55.7%	−2.6%	0.411
**App Usage**				
Ever logged into the app	86.5%	85.3%	−1.3%	0.596
Logged into the app more than once	51.6%	39.9%	−11.8%	0.001 *
Average number of app logins	2.8	2.2	−0.6	0.002 *
Average number of app clicks	34.3	23.3	−11.1	0.000 *
Average number of sections visited (out of six)	3.7	3.4	−0.4	0.030 *
Visited all six sections	45.2%	40.5%	−4.8%	0.169
Average percentage of activities completed ^d^	27.8%	21.9%	−5.9%	0.002 *
**Text Messages**				
Opted out of receiving texts	10.5%	10.1%	−0.3%	0.875
Experienced a bounce back ^e^	25.6%	16.6%	−9.0%	0.001 *
Received a reminder text	61.9%	78.1%	16.3%	0.000 *
Received all content texts	59.1%	68.7%	9.6%	0.003 *
**Sample N**	565	434		

^a^ “Non-Hispanic Other” consists of any participant that did not identify as either Hispanic or Black. Participants in this category identified as White (82%), Asian (13%), American Indian (6%), Native Hawaiian/Pacific Islander (3%), and Other (4%) race. Note: participants could select more than one response. ^b^ “Current hormonal/LARC use” is measured for participants who had ever had sex. ^c^ “Hormonal/LARC use at last sex” is measured for participants who had had sex in the past three months. ^d^ Based on 16 core activities identified by the app developer. ^e^ “Bounce back” is a text message that was sent to a participant but not delivered. * *p* < 0.05.

**Table 3:** Impacts on primary and secondary outcomes at six-week follow-up, for original study [16] and current replication study.

Measure	Total Sample Size	Pulse Intervention	Control	Difference	*p*-Value
**Unprotected sex (in past 6 weeks)**					
Sex without any method					
Original study	1087	22.7	25.1	−2.40	0.265
Replication study	851	23.6	24.5	−0.95	0.694
Sex without a hormonal/LARC method					
Original study	1086	22.1	29.7	−7.56	0.001 *
Replication study	858	28.7	23.8	4.82	0.058
**Contraceptive use**					
Current hormonal/LARC use ^a^					
Original study	763	48.9	49.1	−0.16	0.945
Replication study	571	39.1	36.5	2.60	0.277
Hormonal/LARC use at last sex ^b^					
Original study	578	49.1	51.7	−2.62	0.379
Replication study	409	45.1	40.7	4.40	0.156
**Birth control knowledge (% correct)**					
Original study	1124	51.5	44.5	7.04	0.000 *
Replication study	851	49.8	41.8	8.00	0.000 *
**Attitudes**					
**Attitudes about birth control**					
Disagree that birth control is too much of a hassle to use					
Original study	1122	55.5	53.9	1.61	0.539
Replication study	854	45.5	45.8	−0.33	0.914
Disagree that birth control has too many negative side effects					
Original study	1119	37.5	33.8	3.72	0.144
Replication study	855	23.2	22.0	1.16	0.642
**Attitudes about sexual and reproductive health care**					
Disagree that going to a health care provider for sexual and reproductive health services is hard					
Original study	1120	53.0	51.4	1.70	0.524
Replication study	847	49.9	46.4	3.54	0.265
Disagree that going to a health care provider for sexual and reproductive health services is expensive					
Original study	1119	30.8	25.5	5.30	0.027 ^c^
Replication study	843	29.6	26.0	3.60	0.212
**Self-Efficacy and Intentions**					
**Birth control self-efficacy**					
Confident can use birth control during every sexual intercourse					
Original study	1123	67.3	61.5	5.75	0.025 *
Replication study	850	52.9	51.5	1.43	0.645
**Sexual and reproductive health self-efficacy**					
Confident can go to a health care provider for sexual and reproductive health services					
Original study	1118	80.0	80.3	−0.30	0.898
Replication study	849	75.7	72.6	3.04	0.306
**Intentions**					
Intend to visit a health care provider for sexual or reproductive health services					
Original study	1121	43.4	39.5	3.90	0.120
Replication study	866	42.2	39.6	2.58	0.396
Intend to use a hormonal/LARC method					
Original study	1121	57.2	54.4	2.83	0.273
Replication study	864	54.5	48.6	5.89	0.052

Data were collected post-intervention (six weeks post-baseline). ^a^ “Current hormonal/LARC use” is measured for participants who had ever had sex at baseline. ^b^ “Hormonal/LARC use at last sex” is measured for participants who had ever had sex at baseline and who had had sex in the past six weeks at follow-up. ^c^ *p*-value was not significant after adjusting for multiple hypothesis testing. * *p*-values < 0.05.

**Table 4:** Impacts on primary and secondary outcomes at six-month follow-up, for current replication study.

Measure	Total Sample Size	Pulse Intervention	Control	Difference	*p*-Value
**Unprotected sex (in past three months)**					
Sex without any method	766	31.2	31.9	−0.95	0.694
Sex without a hormonal/LARC method	778	32.0	32.6	−0.53	0.863
**Contraceptive use (among sexually experienced at baseline)**					
Current hormonal/LARC use ^a^	503	37.2	36.7	0.58	0.858
Hormonal/LARC use at last sex ^b^	391	43.4	40.8	2.65	0.508
**Pregnancy history**					
Ever been pregnant	752	16.6	18.3	−1.70	0.278
Pregnancy scare in past six months	751	34.8	33.7	1.15	0.710
**Clinic utilization**					
Visited a provider for sexual and reproductive health services in past six months	784	41.7	38.5	3.18	0.326
**Birth control knowledge (% correct)**	772	50.6	47.1	3.49	0.096

Data were collected six months post-baseline. ^a^ “Current hormonal/LARC use” is measured for participants who had ever had sex at baseline. ^b^ “Hormonal/LARC use at last sex” is measured for participants who had ever had sex at baseline and who had had sex in the past three months at follow-up.

## Data Availability

The data presented in this study are not publicly available due to privacy restrictions. De-identified data are available on request from the corresponding author.
